# High Throughput Screening for Small Molecule Therapy for Gaucher Disease Using Patient Tissue as the Source of Mutant Glucocerebrosidase

**DOI:** 10.1371/journal.pone.0029861

**Published:** 2012-01-17

**Authors:** Ehud Goldin, Wei Zheng, Omid Motabar, Noel Southall, Jae Hyuk Choi, Juan Marugan, Christopher P. Austin, Ellen Sidransky

**Affiliations:** 1 Medical Genetics Branch, National Human Genome Research Institute, National Institutes of Health, Bethesda, Maryland, United States of America; 2 NIH Chemical Genomics Center, National Human Genome Research Institute, National Institutes of Health, Bethesda, Maryland, United States of America; Baylor Research Institute, United States of America

## Abstract

Gaucher disease (GD), the most common lysosomal storage disorder, results from the inherited deficiency of the lysosomal enzyme glucocerebrosidase (GCase). Previously, wildtype GCase was used for high throughput screening (HTS) of large collections of compounds to identify small molecule chaperones that could be developed as new therapies for GD. However, the compounds identified from HTS usually showed reduced potency later in confirmatory cell-based assays. An alternate strategy is to perform HTS on mutant enzyme to identify different lead compounds, including those enhancing mutant enzyme activities. We developed a new screening assay using enzyme extract prepared from the spleen of a patient with Gaucher disease with genotype N370S/N370S. In tissue extracts, GCase is in a more native physiological environment, and is present with the native activator saposin C and other potential cofactors. Using this assay, we screened a library of 250,000 compounds and identified novel modulators of mutant GCase including 14 new lead inhibitors and 30 lead activators. The activities of some of the primary hits were confirmed in subsequent cell-based assays using patient-derived fibroblasts. These results suggest that primary screening assays using enzyme extracted from tissues is an alternative approach to identify high quality, physiologically relevant lead compounds for drug development.

## Introduction

High throughput screening (HTS) is widely used for the identification of small molecule leads that can be developed into pharmacological agents. Assay miniaturization in a 1536 well format has made it possible to screen large numbers of compounds at multiple concentrations in primary screens [1]. However, the optimal conditions for implementing this strategy must be tailored individually for each drug target before implementing HTS.

A number of HTS assays have been performed to identify potential lead compounds for several of the lysosomal storage disorders (LSDs) [2], [3], [4], [5]. Almost all of these screens utilized purified recombinant enzyme as the enzyme source, mainly due to the high specificity of the recombinant enzyme, and the availability of large amounts of the enzyme, since several lysosomal enzyme preparations are currently available for enzyme replacement therapy (ERT). In addition, most lysosomal enzymes are hydrolases, which can be formatted into similar fluorogenic enzyme assays. These conditions enable comparisons between the different screens, ensuring the selection of specific active compounds for a specific enzyme target.

Gaucher disease (GD), the most common LSD, is caused by the deficiency of the lysosomal enzyme glucocerebrosidase (GCase) (EC 3.2.1.45) [6]. The disorder is characterized by a broad spectrum of clinical manifestations, including anemia, thrombocytopenia, massive hepatosplenomegaly, bone disease and in the neuronopathic forms, brain involvement. Treatment options include ERT, substrate reduction therapy (SRT) [7], [8], and, more recently, chaperone therapy utilizing iminosugar derivatives [9]. ERT, infused intravenously at regular intervals, successfully treats many of the systemic manifestations of the disease, and has greatly improved the quality of life for patients with GD [10]. However, studies with both ERT and SRT have shown that these therapies have no impact on neurologic manifestations [11]. Moreover, the cost, especially for the ERT, is prohibitive.

In a previous HTS using recombinant wildtype (WT) GCase, we identified several novel classes of inhibitor molecules with potential chaperone activity, but did not find promising enzyme activators [5]. The lead molecules identified in the screen were further optimized by medicinal chemistry efforts to improve the structure activity relationship (SAR). Some of these compounds were shown to enhance delivery of the enzyme to the lysosome in patient fibroblasts. The potencies of these compounds as small molecule chaperones were generally 100 to 1000-fold weaker than their enzyme inhibitory activities. This discrepancy may result from the differences in assay format used to measure the effect of the compounds, as the patient-derived cells were used in the chaperone assay and the recombinant WT enzyme was used in the original HTS assay. Thus, screening of the library against a mutant form of the enzyme might facilitate the identification of higher quality lead compounds for drug development.

Over 300 mutations in the GCase gene, *GBA1*, have been described [12]. Many cause a single amino acid change, resulting in reduced enzyme activity. The most common mutation in type 1 GD occurs at position c.1226 A>G, causing an N370S amino acid change [13], [14], [15]. Patients with this mutation exhibit variability in phenotype, ranging from asymptomatic individuals to those with severe visceral involvement or bone disease[16]. Thus, the N370S mutant form of the enzyme was selected for the current HTS. In this study we evaluate the use of patient tissue-derived enzyme for HTS and for the validation of lead compounds identified from library screens.

## Materials and Methods

### Reagents and chemicals

Imiglucerase, a recombinant wildtype GCase, was obtained from Genzyme Corporation (Cambridge, MA). N370S recombinant GCase was a gift from Dr. Tim Edmunds at Genzyme. 4-methylumbelliferyl-β-D-glucopyranoside (4MU-β-glc), a blue fluorogenic substrate, resorufin β-D-glucopyranoside (res-β-glc), a red fluorogenic substrate, sodium taurocholate (NaTC), and buffer components were purchased from Sigma-Aldrich (St. Louis, MO). Isofagomine and N- (n-nonyl) deoxynojirimycin (Nonyl-DNJ) were purchased from Toronto Research Biochemicals (Ontario, Canada). Conduritol β-epoxide (CBE) was purchased from Biomol International (Plymouth Meeting, PA). Recombinant SapC (Swiss protein P20097), expressed in E. Coli according to the published sequence, was purchased from Genscript (NJ). A mutant form of the SapC peptide with mutation L349P was generated as well [17].

The NIH Chemical Genomics Center compound library was used for the HTS.

### Spleen tissue preparation

Patient and control spleen samples were obtained from tissue collected at splenectomy or autopsy with written informed consent. The study was approved by the National Human Genome Research Institute Review Board, under permit code: National Insts Hlth - NHGRI - IRB #14. Enzyme extracts from spleen were evaluated as a native source of mutant enzyme for the GCase assay for use in large-scale compound screening. GCase activity was assayed in extracts from spleens from several patients with GD with genotype N370S/N370S. The sample with the highest activity, N370S/N370S-1, was selected as the mutant enzyme preparation for both the HTS and further experiments (Figure S1).

Ninety grams of minced tissue was placed in 600 ml of ice-cold extraction buffer (50 mM Citrate 175 mM KH_2_PO_4_ 0.01% Tween-20 pH 5.9). The tissue was then homogenized using a food blender at maximum speed for 5 minutes, followed by 10 passes in a motor-driven 50 ml glass-Teflon homogenizer (setting 10; nominal clearance, 0.13–0.18 mm). The homogenate was centrifuged at 1000×g for 10 min. The supernatant was passed through a 40 µm filter and aliquots of the resultant spleen homogenate were frozen at −80°C until use.

### Buffers for enzyme assay with spleen extract

The assay buffer consisted of 50 mM citric acid, 115 mM K_2_HPO_4_, 110 mM KCl, 10 mM NaCl, 1 mM MgCl_2_, and 0.01% Tween-20 at pH 5. A solution of 1 M NaOH and 1 M glycine at pH 10.0 was used as the stop solution to increase the fluorescence signal.

### HTS validation in 1536-well plates

In a black 1536-well plate (Greiner Bio-one, Monroe, NC), 2 µl/well of spleen homogenate containing 27 µg protein were incubated with 23 nl of DMSO solution for 5 minutes, followed by the addition of 2 µl/well 4MU-β-glc to a final concentration of 1 mM. After a 30-minute incubation at 37°C, the enzyme reaction was terminated with the addition of 2 µl/well of the stop solution. The fluorescence was then measured in a CCD-based plate reader, ViewLux (Perkin Elmer, Waltham, MA) with an excitation at 365 nm and emission at 440 nm. A BioRAPTR FRD™ Microfluidic Workstation (Beckman Coulter, Inc. Fullerton, CA) was used to dispense reagents into 1536-well plates, and an automated pin-tool station (Kalypsys, San Diego, CA) was used to transfer 23 nl/well of compound to the assay plate. Nonyl-DNJ, a known GCase inhibitor, was used as a control compound in this assay.

The initial assay, tested in the 1536-well plate format with the solvent DMSO, showed a signal-to-basal ratio of 20-fold, a CV of 6.3% and a Z′ factor of 0.91. Subsequently, all compounds were screened at seven concentrations (1∶5 serial dilutions) [1], [18]. The active compounds from the qHTS were selected based on their EC_50_/IC_50_ values and concentration response curves.

### Validation of the ability of selected compounds to chaperone in a cell based assay

Patient fibroblasts were seeded in 96 well glass bottom plates (Greiner Monroe, NC). After 48 hours they were treated with five compounds identified from the screening assay using N370S/N370S spleen homogenate and NCGC00092410, which was identified from the recombinant WT screen. In addition, cells were treated with isofagomine and Nonyl-DNJ as positive controls and DMSO as a negative control. The cells were treated with six different concentrations of drug (from 1 nm to 50 µM) for five days, with one replacement of media. Some compounds were found to be toxic at the highest concentration. Cells were fixed with 3% paraformaldehyde and stained with a nuclear marker (Hoechst Invitrogen, Carlsbad, CA), the lysosomal marker LAMP2 (H4B4 Abcam, Cambridge, MA), and anti-GCase (in house antibody Rabbit 386). Regions of interest (ROI) were determined by LAMP2 localization in 4 fields/well, and the intensity of GCase staining was measured in the ROI using an automatic fluorescent microscope (BD biosciences, Sparks, MD) for each compound and for each cell culture tested.

## Results

### Recombinant N370S GCase has a higher pH optimum and a lower V_max_ than wildtype GCase

**Figure 1:**
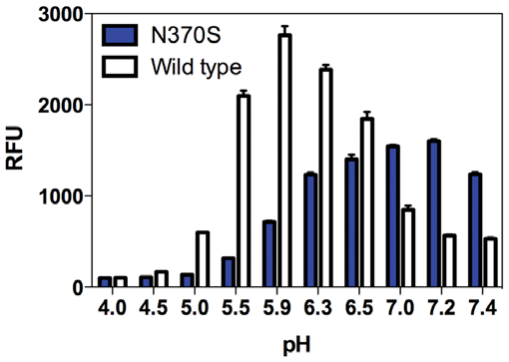
pH profiles of recombinant N370S and WT GCase. The assay was performed in the presence of 10 mM NaTC. While the WT enzyme has a pH optimum of 5.9, the N370S enzyme has a broad optimum of around pH 7.2.

The activity of the recombinant N370S mutant enzyme was first compared to WT enzyme. We found that the pH optimum of the mutant enzyme was 7.2, versus 5.9 for the WT enzyme (Fig. 1).

The N370S enzyme also had a reduced V_max_ toward two different fluorogenic (red and blue) substrates when assayed in the presence of NaTC at pH 5.9, the optimal pH for WT enzyme. The V_max_ values were 11.7 pmole/min and 41.9 pmole/min and the Km values were 33.3 µM and 31.3 µM for the mutant and WT enzyme, respectively. The mutant enzyme activity was 27.9% of the WT enzyme as calculated by the V_max_ values. However, the activity of the mutant enzyme was significantly lower at acidic pH, and was virtually absent at pH 5, the normal lysosomal pH.

**Figure 2:**
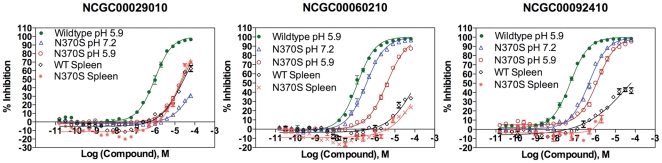
Concentration responses of three GCase inhibitors determined using recombinant WT GCase, recombinant N370S mutant enzyme, WT GCase from normal spleen extract and N370S mutant enzyme from a spleen of a patient with GD (genotype N370S/N370S). The three inhibitors were identified from the previous HTS using recombinant WT GCase.

The activities of three inhibitors identified in the previous HTS [19] were tested with recombinant WT and N370S GCase, as well as spleen tissue extracts from controls and patients with genotype N370S/N370S. In the recombinant N370S enzyme assay, all three GCase inhibitors exhibited a 24- to 31-fold reduction in activity, as compared with the recombinant WT enzyme assay (Fig. 2). The inhibitory activities were further reduced (>700-fold) for two of the inhibitors, NCGC00092410 and NCGC00060210 in the assays using WT and N370S spleen extracts. The results demonstrated that the inhibitors exhibited very different activities with the different enzyme preparations tested.

### WT and mutant spleen GCase have a lower and identical pH optimum, and are less dependent on NaTC

**Figure 3:**
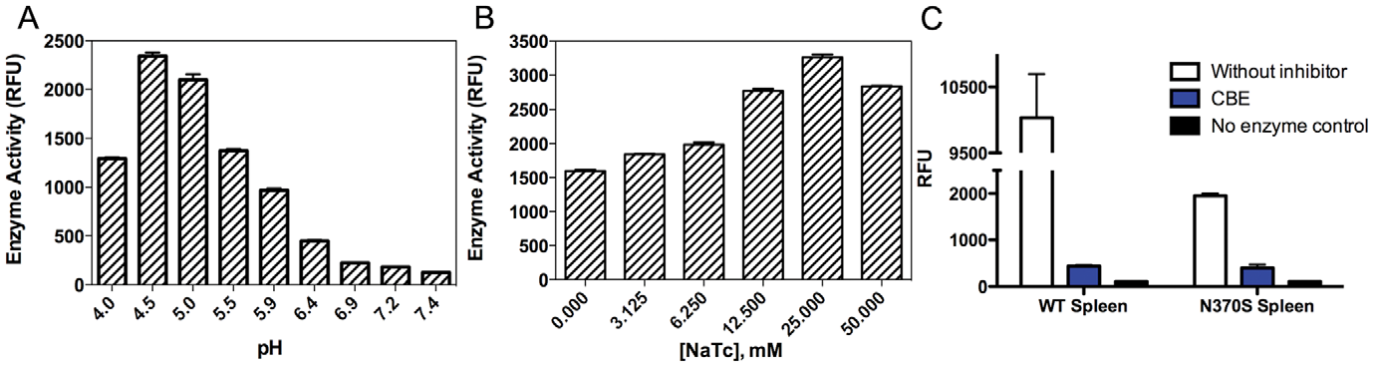
Characterization of spleen GCase activity. A. pH optimum, B. NaTC dependency, C. Inhibition of spleen GCase activity by CBE.

**Figure 4:**
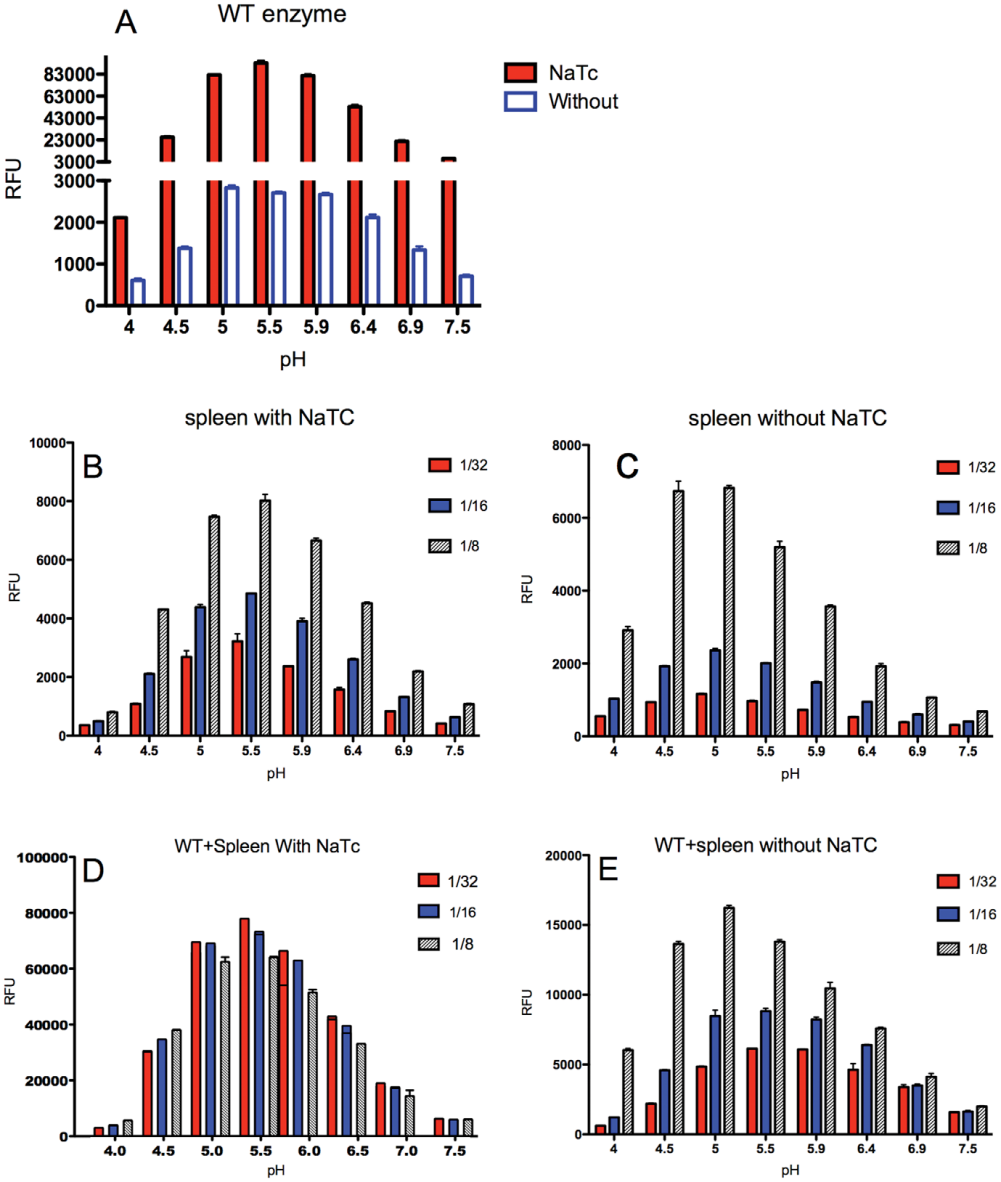
Effect of adding the spleen preparation on the activity of WT recombinant GCase. pH dependency and effect of sodium NaTC on the recombinant enzyme activity were assayed. A. activity of WT recombinant enzyme with and without NaTC. B and C. Different dilutions of N370S spleen in the presence (B) or absence (C) of NaTC. D. and E. WT recombinant enzyme mixed with increasing concentrations of spleen extract with (D) and without (E) NaTC.

The pH optimum of endogenous GCase in the WT spleen extract was below 5 (Fig. 3a). A similar profile was obtained for the N370S spleen (Fig. 4c). The enzyme activity of both the WT and mutant spleen extract had a lower dependence on NaTC than WT recombinant enzyme, with about 2-fold activation compared to more than 20-fold respectively (Figs. 3b, 4a). In order to discriminate between GCase and other glycosidase enzymes capable of cleaving the substrate, spleen preparations were treated with the GCase-specific inhibitor conduritol β-epoxide (CBE) [20]. In both WT and N370S spleen extracts, the amount of activity not inhibited by CBE at pH 5 was minimal, indicating that the other glucose hydrolases do not play a major role in this assay using the rough enzyme preparation (Fig. 3c).

The V_max_ of the endogenous mutant GCase from N370S spleen was lower than that from WT spleen, with values of 9.3 and 22.6 pmol/mg protein/min respectively, while the Km was similar, 1917 µM for N370S and 1519 µM for WT spleen enzyme. Thus, the deficient GCase activity in lysosomes in N370S/N370S patients may result from both the reduced amount of mutant protein delivered to the lysosome, and the decreased catalytic ability of the mutant enzyme.

### Recombinant WT GCase adopts the characteristics of spleen enzyme when it is spiked into the spleen preparation

The lower pH optimum of spleen-derived GCase compared to recombinant enzyme, and its lack of activity-dependence on NaTC suggested that the spleen preparations have other native components critical for optimal enzyme activity. To further demonstrate this hypothesis, we added WT recombinant enzyme to N370S spleen extract with and without NaTC before the measurement of the GC activity. Without NaTC, the activity of the recombinant WT enzyme was only 5% of that seen in the presence of NaTC but its pH optimum shifted to ∼5 (Fig. 4a). The GCase activity in the N370S spleen extract remained the same in the presence or absence of NaTC. (Figs. 4b,c). The pH optimum was also lower without NaTC. Moreover, the GCase activity of the N370S spleen preparation increased further when the concentration of spleen extract increased from 1/32 to 1/8 without NaTC (7-fold), compared with NaTC (2.5-fold), at optimal pH (Figs. 4b,c). In the final step of this experiment, recombinant WT enzyme, which was dependent on NaTC (Fig. 4a), was added to the N370S spleen extract and the activity determined. Figures 4d,e show the activity of WT GCase after subtracting the contribution of endogenous N370S GCase (Figs. 4b,c). In the presence of N370S spleen extract without NaTC, the pH optimum of recombinant WT enzyme was also lowered from 5.9 to 5, and the activity was dependent on the concentration of the spleen extract (Fig. 4e). In contrast, in the presence of NaTC, the activity was not dependent on the concentration of spleen extract (Figs. 4d,e). Similar results were obtained using the recombinant N370S enzyme, where the pH optimum was reduced from neutral to approximately 5. Thus, while the parameters of recombinant GCase activity measured in the presence of the spleen extract resembled those of the spleen homogenate, the spleen component involved in activating the enzyme was obscured by the NaTC.

### SapC and PS are the critical components found in GCase preparations from spleen

The difference in the activity profile of recombinant enzyme in the presence of spleen homogenate, compared to the same enzyme in assay buffer, raised the possibility that other factors in the spleen homogenate compensated for the enzyme activation property of NaTC. We explored the role of SapC, a natural cofactor of GCase. SapC was previously shown to compete with NaTC in activating GCase [21].

**Figure 5:**
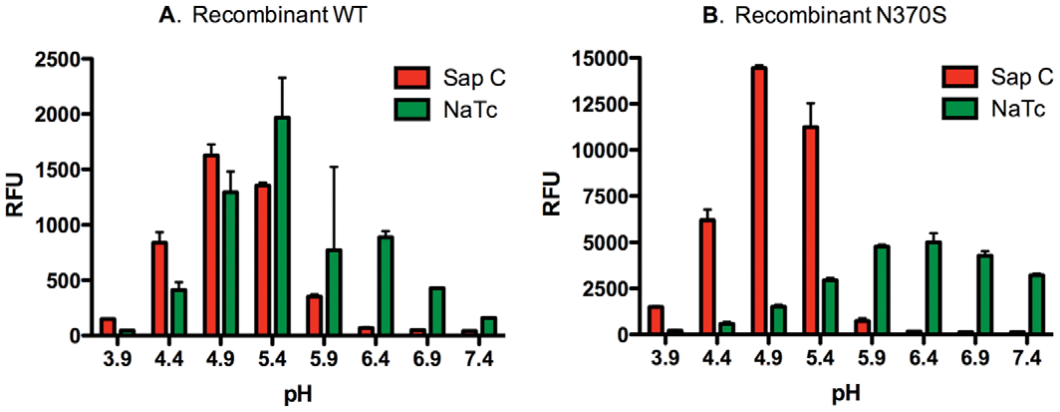
Effect of SapC versus NaTC on the activity of WT (A) and N370S recombinant (B) GCase. In the presence of SapC, the activity of both WT (A), N370S (B) enzyme gave a sharp peak near pH 5, compared to the broader peak and higher pH seen using NaTC.

The enzyme activities of recombinant WT (5 nM) and N370S GCase (100 nM) were tested in the presence of SapC and phosphatidyl-serine as described [22]. We found that the enzyme activity was eight-fold higher in the presence of SapC than the basal level. The maximal enzyme activity in the presence of SapC was higher than that obtained in the presence of 10 mM NaTC (Fig. 5). While both enzymes showed activity at a wide pH range in the presence of NaTC, their peak activity in the presence of SapC was narrowed to around pH 5 with very little activity at neutral pH. The effect of mutant SapC on activation of these enzymes was limited (data not shown). The results indicated that in the presence of SapC and phosphatidyl-serine, the pH optimum and enzyme activity of both recombinant GCases were similar to those of the spleen homogenate.

### A compound screen using GCase from the spleen preparation yields both activators and inhibitors of GCase

A library of 250,000 compounds was screened at five distinct compound concentrations using mutant N370S GCase from spleen and the fluorogenic substrate 4MU-β-glc. The complete results of this screen were deposited in PubChem (AID 2101) [23]. In order to eliminate the false positive compounds, the primary screen results were first compared to those from two other previous screens for alpha-glucosidase and alpha-galactosidase using similar fluorogenic substrates [2], [3]. Ultimately, a set of 30 lead GCase enzyme activators and 14 potent enzyme inhibitors were identified as primary active compounds meriting further validation (Table 1). Five of these compounds were selected for further validation as a proof-of principle and are described in Table 2.

**Table 1:** Classification of active compounds identified from the screen assay using the N370S mutant enzyme in spleen tissue.

Activity	Distribution	Curve Classification*										
		1.1	1.2	1.3	1.4	2.1	2.2	2.3	2.4	3	4	5
Activation	# of compounds	**30**	212	0	95	526	2116	0	421	1135	237353	519
	% of library	0.01%	0.09%	0.00%	0.04%	0.21%	0.86%	0.00%	0.17%	0.46%		
Inhibition	# of compounds	**14**	75	0	344	22	1175	0	242	1394	96.61%	0.21%
	% of library	0.01%	0.03%	0.00%	0.14%	0.01%	0.48%	0.00%	0.10%	0.57%		

*Criteria for classification of compounds are described in table S2.

**Table 2:** Compounds discovered in the spleen qHTS tested for modulation of GCase activity and GCase translocation in cell assays.

Compound	Series	Effect	Potency	
			Enzyme assay*	Translocation**
			(micromolar)	
MLS000674724	Hydroxidiasole	Inhibitor	0.4	−−
NCGC00182292	Quinazoline	Inhibitor	0.25	+−
NCGC00159568	Thiourea	Inhibitor	0.31	++
NCGC00182186	Carboxyamide	Activator	3	++
NCGC00182510	Benzoate	Inhibitor	1	+−

*As measured on spleen enzyme preparation.

**Translocation experiments performed on WT, N370S/N370S, L444P/L444P and L444P/Rec*Nci1* fibroblasts.

### Cell based assays confirm the chaperone activity of GCase activators and inhibitors

**Figure 6:**
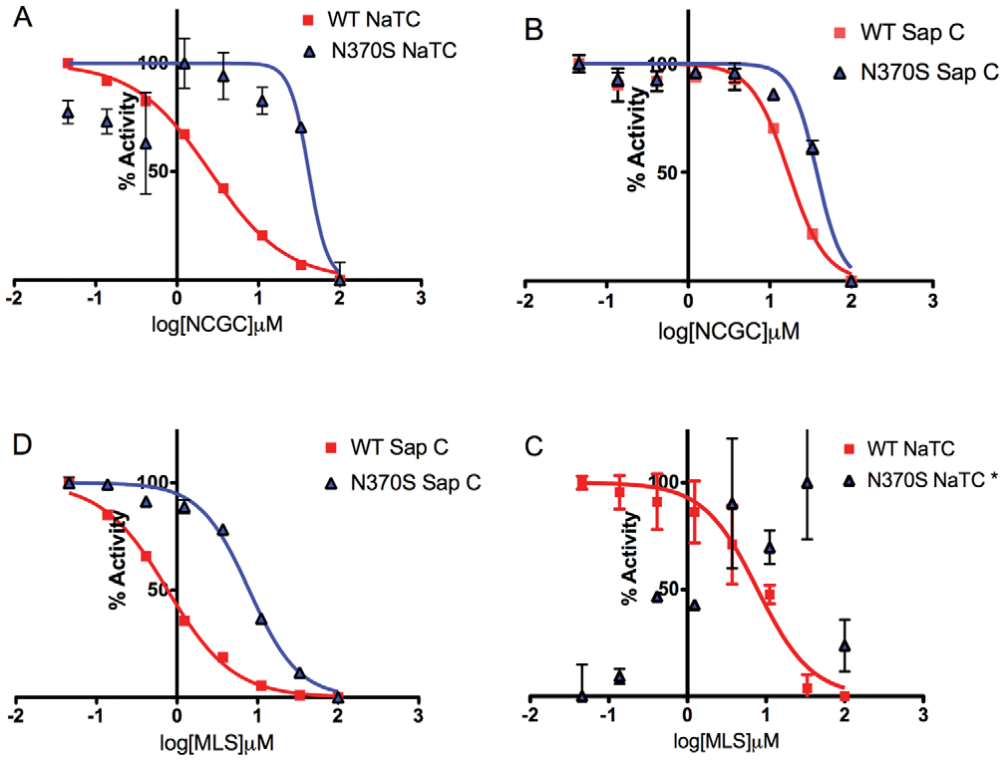
Dose response increase in lysosomal content of GCase in fibroblasts treated with selected identified compounds. Cells were treated with six drug concentrations (1 nM to 50 µM) for 5 days. The intensity of GCase staining in the lysosomes was measured using an automatic fluorescent microscope. The slope of dose dependent increase in fluorescence was plotted and statistically significant slopes are labeled with (*). Some compounds, including the activator NCGC00182510, were able to increase the lysosomal content of both N370S and L444P mutant enzymes. Some compounds were found to be toxic at the highest concentration and that point was removed from calculations.

Fibroblasts from patients with GD having the following genotypes: N370S/N370S (severe visceral phenotype), N370S/N370S (mild phenotype), L444P/L444P, and L444P/Rec*Nci*1, were incubated for 5 days with the selected active compounds identified in the primary screen, as well as compounds known to have GCase chaperone activity. The intensity of GCase staining in lysosomes was determined using automatic fluorescence microscopy. The slope of the dose dependent increase in fluorescence above the DMSO baseline was calculated using linear regression (Figure 6), and slopes with statistical significance (P<0.05) are indicated. Some compounds, including the activator NCGC00182186, were able to increase the lysosomal content of both N370S and L444P mutant enzymes. In a parallel experiment, the effect of these compounds on GCase activity was tested in the same cells (Table 2). In addition, compounds were tested for binding properties as well as their effect on the thermal stability of the recombinant enzyme (not shown). Several of the active compounds discovered in the qHTS were chosen for SAR studies and optimized compounds continue to be subjected to further preclinical studies [24].

### HTS using N370S spleen enzyme yields more activators and fewer inhibitors than identified using recombinant GCase

**Figure 7:**
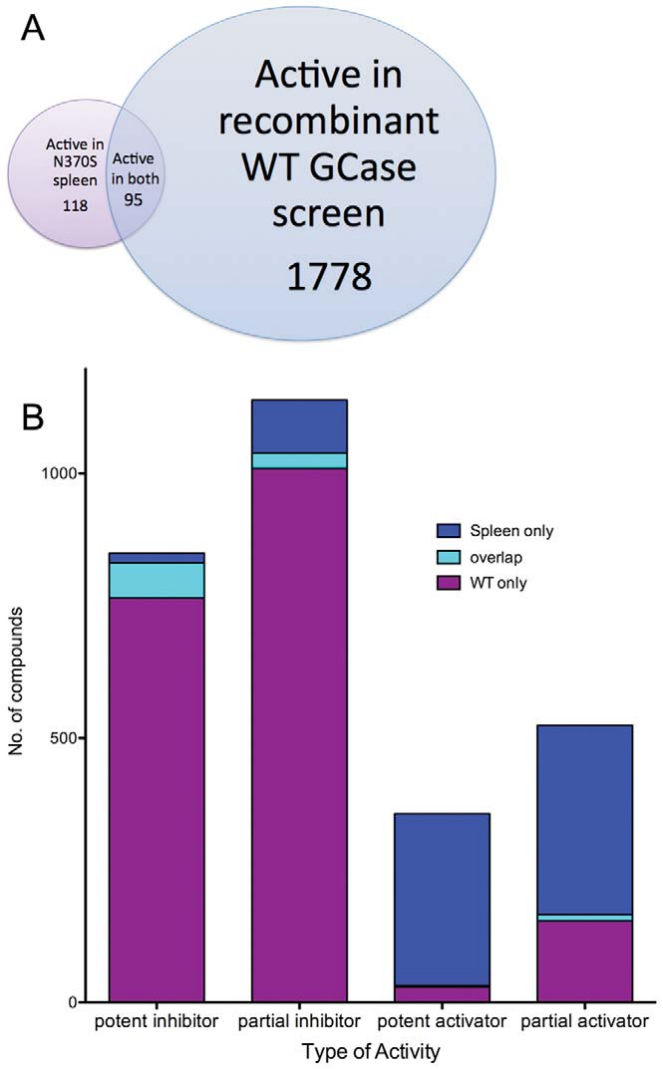
Comparison of the active compounds identified in the WT and N370S HTS experiments. A. Distribution of active compounds in the WT (blue) and N370S/N370S spleen (red) HTS experiments. B. Overlap between active compounds tested in both experiments by type of activity.

To compare the results of primary screens using the N370S spleen enzyme with those from recombinant WT GCase, we analyzed the 52,000 compounds that were screened using both enzyme preparations. The primary screen with GCase from N370S spleen tissue yielded a relatively smaller number of active molecules, and only about 8% of the total number of hits found in the screen using the recombinant WT enzyme were identified in the current screen (Fig. 7a). Approximately 92% of potent inhibitors identified in the initial screen with the recombinant WT enzyme were not detected in the current screen using the spleen enzyme extract (Fig. 7b, Table S1). In addition, about 97% of robust activators identified in the current screen using enzyme extract from N370S spleen, were not detected in the previous screen using recombinant WT enzyme. Most of the newly identified hits in the screen with N370S mutant enzyme were activators of the mutant enzyme. A few compounds that showed activator properties in the current screen but were identified as “inhibitors” in the previous screen using the WT recombinant enzyme were eliminated based on their fluorescence and non-selective properties.

### The difference in the results of the two screens is attributed to the presence of Sap C and PS in the spleen preparation

**Figure 8:**
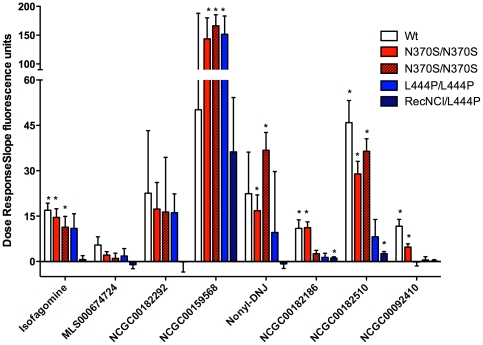
Potency of selected compounds in the WT and N370S mutant enzyme assay with NaTC or SapC. Compound NCGC0092410 (NCGC), discovered in the recombinant WT HTS, inhibited the WT enzyme much better than N370S in NaTC (A), but showed less inhibition of WT enzyme in the SapC assay (B). Compound MLS000393962 (MLS) (C,D), identified in the N370S/N370S spleen HTS, inhibited the WT recombinant enzyme, and had no effect on the recombinant N370S enzyme with the addition of NaTC (C). However, in the presence of SapC (D), there was an enhanced inhibitory effect on the WT enzyme as well as the N370S enzyme. * No trendline fit possible.

We then selected a set of compounds and evaluated their activities in the presence of SapC or NaTC using the different enzyme preparations (Fig. 8). Some of the compounds exhibited relatively high inhibition of GCase activity with the recombinant enzyme in the presence of NaTC, but much weaker inhibition in the assay using enzyme extract from spleen tissue (Fig. 8a). Their inhibitory activities to the recombinant enzyme were reduced significantly when SapC replaced NaTC in the enzyme assay (Fig. 8b). On the other hand, some of the activator compounds that were more active with the N370S enzyme extract from spleen showed much weaker or no activity with the recombinant enzyme in the presence of NaTC (Fig. 8c). These compounds became more active in the same recombinant enzyme assay when NaTC was replaced with SapC (Fig. 8d). These results indicate that the activities of some of the compounds in the GC assay with the WT recombinant enzyme were likely dependent on NaTC, which may explain the reduced activity in the cell-based assays.

## Discussion

The use of recombinant WT proteins for high throughput screening is widely employed, and often leads to the identification of novel enzyme inhibitors. However, in the case of lead compound discovery for GCase, we sought to explain the weaker activities in cell-based assays testing lead compounds previously identified in screens using recombinant WT enzyme. We hypothesized that native cofactors of GCase absent from the recombinant enzyme preparation might be responsible for the discrepancy in the activities of these inhibitors, and that this might be corrected by an alternative screening strategy using enzyme extracted from patient tissues. In addition, the screen with recombinant WT GCase did not yield candidate enzyme activators, suggesting that screens using WT enzyme might not be appropriate for the detection of activators.

We found that some previously identified inhibitors had reduced potency when tested in the assay using enzyme extracts from WT and patient tissues. This suggested that GCase screening using patient tissue extracts could identify a different set of active compounds from the primary screen.

The initial HTS assay was adopted from the clinical diagnostic procedure for determining GCase activity in patient samples. This assay requires NaTC in the buffer to activate the enzyme. While this assay format is useful for the diagnosis of GD, it has limitations when used for compound screens. First, this assay is performed at pH 5.9, which is the optimal pH for the enzyme reaction in the presence of NaTC. However, the lysosome usually has a pH of 4.5 to 5. Thus, the enzyme may have different conformations in these different pH environments, resulting in different sensitivities to small molecules. Second, essential native co-factors of GCase, including SapC and phosphatidyl-serine, are missing in the purified recombinant enzyme mix, and their absence may also change the enzyme conformation significantly. Third, a combination of recombinant WT enzyme and high concentrations of NaTC serve to optimize the enzyme assay to a high activity level, which may prevent the identification of activators from the compound library screen. Lastly, mutant enzyme should be used because it is the actual drug target.

Our studies show that enzyme extracts from WT and patient tissues had a pH optimum of 5, closer to the lysosomal pH (Fig. 3). We then found that when the recombinant enzyme was tested in the presence of a small amount of spleen homogenate, it acquired the characteristics similar to the enzyme extract from spleen, including a reduced dependence on NaTC and a lower pH optimum (Fig. 4). These results indicated that enzyme extract from spleen contains native cofactors absent from the recombinant enzyme. Stimulating the activity of the recombinant enzyme with NaTC shifts the pH optimum to 5.9, which is above lysosomal pH.

We found that recombinant WT enzyme acquired characteristics similar to the native enzyme when the NaTC in the buffer was replaced by phosphatidyl-serine and SapC (Fig. 5), suggesting that the higher pH optimum in the previous assay was due to the addition of NaTC. With recombinant N370S enzyme, the pH optimum shifted from 7 in the presence of NaTC to 5 when phosphatidyl-serine and SapC replaced NaTC. An increased pH optimum was also noted in cultured fibroblasts from patients with genotype N370S/N370S in the presence of NaTC [25]. Thus, the inclusion of NaTC in the buffer may impact the effect of compounds on enzymatic activity, and subsequently the nature of compounds identified from library screens. For example, a compound that interacts with the head-group of taurocholate may inhibit the enzymatic activity by reducing the enzyme activation by NaTC. The active conformation of GCase in the presence of NaTC may be significantly different from the enzyme in the presence of the physiological cofactors phosphatidyl-serine and SapC. The enzyme inhibitors could have different affinities to these two different active conformations of GCase (i.e. in the presence of native co-factors or in the presence of NaTC). These may have contributed to the discrepancy in inhibitory activities found between the assay with recombinant enzyme in the presence of NaTC and that using enzyme extract from spleen tissue (Fig. 7).

The N370S mutant enzyme was selected for compound screening because it is among the most common Gaucher mutations. Moreover, since the V_max_ value of the N370S mutant form of the enzyme is reduced, it can facilitate the detection of small molecule compounds that can increase activity of this mutant enzyme. Thus, the compound library screens can target small molecules that increase the V_max_ of N370S mutant enzyme and thus enhance enzyme activity in late endosomes and lysosomes [26].

The use of recombinant mutant enzyme for HTS might seem advantageous because the target protein is pure and the enzyme specificity is high. However, the activation of a non-native enzyme confirmation by NaTC could lead to another set of false positive compounds that may be less active later in confirmatory cell-based assays. Thus, for the current screen of 250,000 compounds, we used the enzyme extract from spleen of a patient with GD (genotype N370S/N370S). Frozen tissue samples from patients with GD harboring mutations such as N370S are readily available because, in the past, many patients underwent splenectomies to reduce their disease burden. The significant residual activity of GCase associated with this mutant form of the enzyme was an asset in the screen assay development. While nonspecific compounds were also found in the screen due to the use of this rough preparation, they were readily eliminated later in the secondary and tertiary screens with cell-based assays (Fig. 6).

Structurally, the WT and N370S forms of GCase are quite similar [27], and hence, one might expect similar results from compound library screens for the two enzymes. However, because of the reasons discussed above, the HTS using enzyme extract from the N370S spleen yielded a new set of active compounds not found using recombinant WT enzyme. Moreover, certain active compounds previously found in the screen using recombinant WT enzyme had far lower activities in the assay using enzyme extract from spleen, suggesting that NaTC might interact with these compounds or alter the enzyme's confirmation (Fig. 8). Thus, the use of enzyme extract from patient tissue in the primary screen may identify new lead compounds not identified in screens using recombinant enzymes.

The screen with mutant enzyme also improved our ability to discover enzyme activators, especially those enhancing the activity of mutant enzyme. Compounds with activation properties were found at a 3.6-fold higher rate in the current screen than that in the previous screen using recombinant WT enzyme. Selective activators of the enzyme are usually allosteric binders. These enzyme activators with chaperone activity should better assist the movement of mutant proteins from the ER to the lysosome, because they do not need to be released from the enzyme in the lysosome to hydrolyze the substrate. This mechanism of action differs from enzyme inhibitor-based chaperones, where the inhibitors must be released from the mutant proteins in the lysosome before the enzyme can be functional. Thus, the dosing of activator-based chaperone therapy is much simpler, facilitating preclinical and clinical development.

The results observed when SapC replaced NaTC in the assay using recombinant enzyme indicate that both the WT and mutant forms of the enzyme have a similar affinity to the compounds discovered in the HTS using enzyme extract from spleen. This suggests that the difference between the two enzymes is primarily the decreased V_max_ value in the N370S mutant enzyme. Thus, activators that can increase the V_max_ value of N370S enzyme might be useful for the treatment of other mutant forms of GCase. It has been reported that with deoxynojirimycin, an inhibitor-based iminosugar chaperone compound developed for the treatment of Pompe disease, only a fraction of mutant forms of α-glucosidase responded to the treatment [28]. In contrast, activators bind in a location distant from the active site, and therefore may be useful for a wide range of mutations.

In conclusion, this study is the first utilizing enzyme extract from patient tissue for HTS of compound libraries to identify enzyme activators and inhibitors. Several of the lead compounds identified are currently being developed and tested as possible therapies for patients with Gaucher disease. This new screening strategy can also be applied to lead discoveries for other diseases where frozen tissue specimen may be readily available. The use of enzyme extracts from patient tissues has several advantages for HTS including relatively easier enzyme preparation (especially for mutant enzymes), a physiologically relevant enzyme environment for better lead discovery, and a much simplified screen assay over the cell-based assays. In addition, it is likely that more activators will be found using the enzyme extracts derived from patient tissues and the compound activities in this assay will correlate better later with those in cell-based confirmatory assays. Therefore, adaptation of this new screening strategy may greatly facilitate lead discovery as well as the “hit to lead” progression in the early drug discovery process.

## Supporting Information

Figure S1 GCase activity assayed in patient spleen homogenates. K_cat_ is the V_max_ value of the enzyme divided by the protein concentration. Differences in activity may represent individual differences or tissue preservation issues.
(DOC)

Table S1 Comparison of active compounds identified in the two GCase screens. The numbers of compounds found active in the recombinant enzyme screen (columns) and in the N370S spleen screen (rows) are presented by type of activity. The numbers of compounds active in only one screen are presented in the last column and the last row. Clearly the overlap of active compounds in the two screens is miniscule. Compounds with opposite activities in the two screens were tested individually and found to have autofluorescence.
(DOC)

Table S2 Criteria for classification of compounds. Compounds are evaluated by their activity/concentration curves following qHTS.
(DOC)
