# Supplementary material for: High Throughput Screening for Small Molecule Therapy for Gaucher Disease Using Patient Tissue as the Source of Mutant Glucocerebrosidase
Source: PLoS One. 2012 Jan 17;7(1):e29861. doi: 10.1371/journal.pone.0029861 (PMC3260169; doi:10.1371/journal.pone.0029861)
Supplement: Figure S1 — GCase activity assayed in patient spleen homogenates. Kcat is the Vmax value of the enzyme divided by the protein concentration. Differences in activity may represent individual differences or tissue preservation issues. (DOC) [file pone.0029861.s001.doc]

**Figure S1**. GCase activity assayed in patient spleen homogenates. K_cat_ is the V_max_ value of the enzyme divided by the protein concentration.
