# Supplementary material for: High Throughput Screening for Small Molecule Therapy for Gaucher Disease Using Patient Tissue as the Source of Mutant Glucocerebrosidase
Source: PLoS One. 2012 Jan 17;7(1):e29861. doi: 10.1371/journal.pone.0029861 (PMC3260169; doi:10.1371/journal.pone.0029861)
Supplement: Table S1 — Comparison of active compounds identified in the two GCase screens. The numbers of compounds found active in the recombinant enzyme screen (columns) and in the N370S spleen screen (rows) are presented by type of activity. The numbers of compounds active in only one screen are presented in the last column and the last row. Clearly the overlap of active compounds in the two screens is miniscule. Compounds with opposite activities in the two screens were tested individually and found to have autofluorescence. (DOC) [file pone.0029861.s002.doc]

**Supplemental Table 1**. Comparison of active compounds identified in the two GCase screens

| **Recombinant wildtype GCase** | | | | | | |
| --- | --- | --- | --- | --- | --- | --- |
| **N370S Spleen GCase** |  | Robust inhibitor | Partial inhibitor | Robust activator | Partial activator | Inactive |
|  | Robust Inhibitor | 26 | 12 | 0 | 0 | 18 |
|  | Partial inhibitor | 40 | 17 | 0 | 0 | 100 |
|  | Robust activator | 1 | 3 | 1 | 6 | 325 |
|  | Partial activator | 5 | 5 | 2 | 6 | 358 |
|  | Inactive | 765 | 1010 | 29 | 154 | 50410 |
