# Supplementary material for: High Throughput Screening for Small Molecule Therapy for Gaucher Disease Using Patient Tissue as the Source of Mutant Glucocerebrosidase
Source: PLoS One. 2012 Jan 17;7(1):e29861. doi: 10.1371/journal.pone.0029861 (PMC3260169; doi:10.1371/journal.pone.0029861)
Supplement: Table S2 — Criteria for classification of compounds. Compounds are evaluated by their activity/concentration curves following qHTS. (DOC) [file pone.0029861.s003.doc]

**Supplemental Table S2** Criteria for classification of compounds.

| **Curve Class** | **Asymptotes** | **r^2^** | **Efficacy** | **Description** |
| --- | --- | --- | --- | --- |
| 1 | Higher and lower | ≥ 0.9 | > 80% | 1.1: Complete curve; high efficacy |
|  |  |  | Min - 80% | 1.2: Complete curve; partial efficacy |
|  |  | < 0.9 | > 80% | 1.3: Complete curve; high efficacy; poor fit |
|  |  |  | Min - 80% | 1.4: Complete curve; partial efficacy; poor fit |
| 2 | Lower only | ≥ 0.9 | > 80% | 2.1: Partial curve; high efficacy |
|  |  |  | Min - 80% | 2.2: Partial curve; partial efficacy |
|  |  | < 0.9 | > 80% | 2.3: Partial curve; high efficacy; poor fit |
|  |  |  | Min - 80% | 2.4: Partial curve; partial efficacy; poor fit |
| 3 | Lower only |  | >Min | 3: Single point of activity |
| 4 |  |  | < Min | 4: Inactive |
| 5 |  |  | >Min | 5: Inconclusive |
